# Case Report: Necrotizing pneumonia in pediatric patients: a rare case of unilateral necrosis in two entire lobe tissues

**DOI:** 10.3389/fped.2024.1428402

**Published:** 2025-01-30

**Authors:** Jingli Zhang, Yingqian Zhang, Longfei Gao, Huifang Wu, Xiaojuan Liu, Fan Yang, Yingxue Wang

**Affiliations:** Third Department of Respiratory, Hebei Children’s Hospital, Shijiazhuang, Hebei, China

**Keywords:** necrotizing pneumonia, *Mycoplasma pneumoniae*, pediatric, radiographic findings, unilateral necrosis of the entire two-lobed lung

## Abstract

**Background:**

Necrotizing pneumonia is a serious complication of *Mycoplasma pneumoniae* infection, especially in pediatric patients. Although necrotizing pneumonia is a rare condition, the occurrence of unilateral necrosis in two entire lobe tissues is even rarer. This case study presents a 5-year-old male child with necrotizing pneumonia caused by *Mycoplasma pneumoniae*, describing the clinical features, laboratory abnormalities, and radiographic findings associated with this condition, particularly the rare occurrence of unilateral necrosis in two entire lobe tissues.

**Case presentation:**

The patient presented with a 14-day history of fever and cough, accompanied by paroxysmal cough with continuous sounds, congested throat, increased respiratory effort, and abnormal lung findings on physical examination. Laboratory investigations revealed elevated white blood cell count, increased inflammatory markers, elevated liver enzymes, coagulation dysfunction, and hypoalbuminemia. Imaging studies showed the presence of pleural effusion and progressive necrotizing pneumonia, including the rare occurrence of unilateral necrosis affecting both entire lobe tissues.

**Conclusion:**

Necrotizing pneumonia caused by *Mycoplasma pneumoniae* infection can present with prolonged fever, elevated inflammatory markers, pleural effusion, and progressive necrosis of lung tissue, with the occurrence of necrosis observed unilaterally in both entire lobe tissues is even rarer. Monitoring D-dimer levels is essential for evaluating the possibility of necrotizing pneumonia. Early recognition and appropriate management are crucial to improve outcomes in these patients.

## 1 Introduction

Necrotizing pneumonia is a severe complication of *Mycoplasma pneumoniae* infection, particularly in pediatric patients. It is characterized by progressive necrosis of lung tissue and can lead to significant morbidity and mortality. Although necrotizing pneumonia is a rare condition, the occurrence of unilateral necrosis in two entire lobe tissues is even rarer. Understanding the clinical and radiographic features of this condition is crucial for early recognition and appropriate management. This article aims to present a case study of a 5-year-old male child with necrotizing pneumonia caused by *Mycoplasma pneumoniae* infection, highlighting the clinical manifestations, laboratory findings, and imaging characteristics associated with this condition, particularly the rare occurrence of unilateral necrosis in two entire lobe tissues.

## 2 Case description

**Figure 1:**
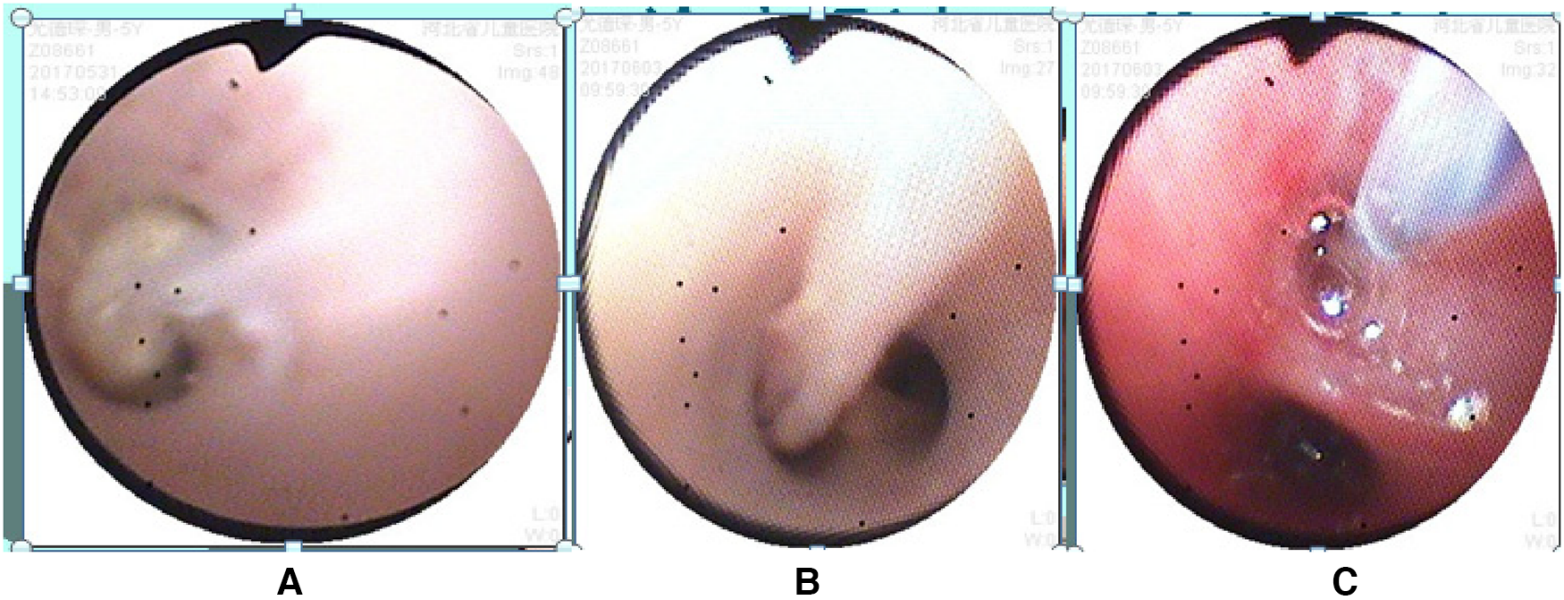
Bronchoscopy findings: **(A,B)** mucous plugs observed in the bronchi, **(C)** bronchial brushing procedure for treatment. Annotation: The bronchoscope is in the close-up mode, magnifying 45 times at a distance of 3 mm from the tip.

**Figure 2:**
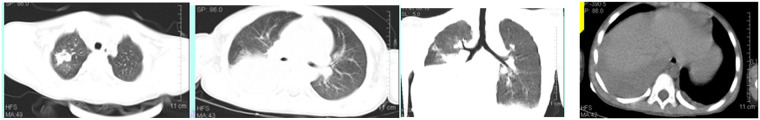
Pulmonary CT findings on Day 7st of onset.

**Figure 3:**
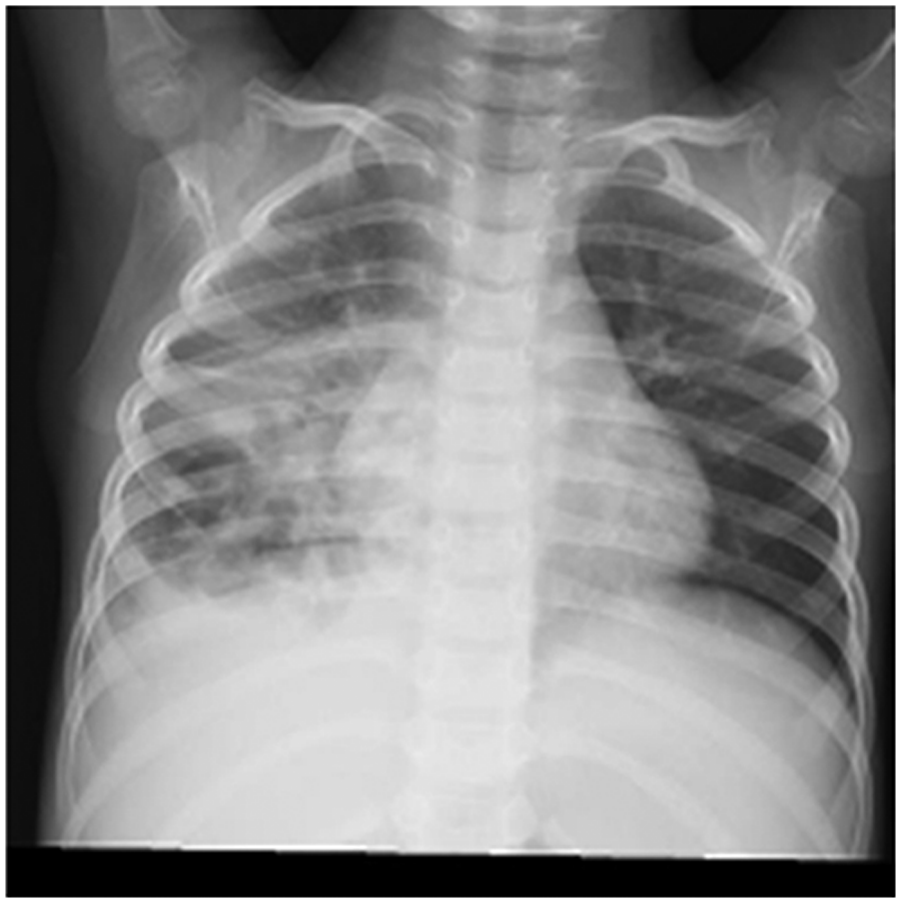
Chest x-ray findings on 21st of onset.

**Figure 4:**
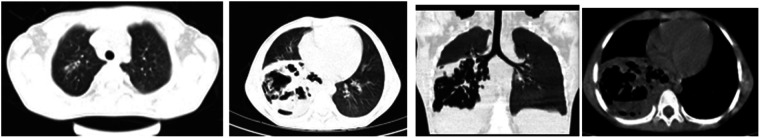
Pulmonary CT findings on Day 21st of onset.

**Figure 5:**
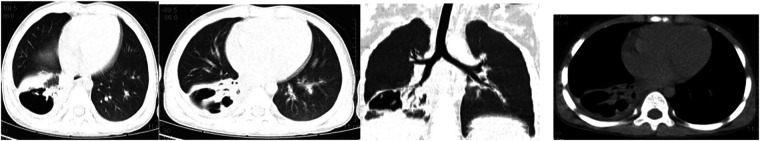
Pulmonary CT findings on Day 44st of onset.

**Figure 6:**
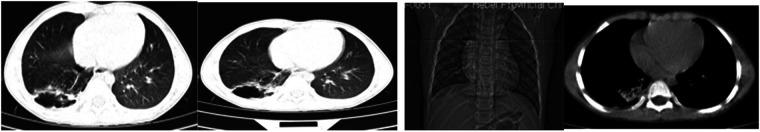
Pulmonary CT findings on Day 72st of onset.

**Figure 7:**
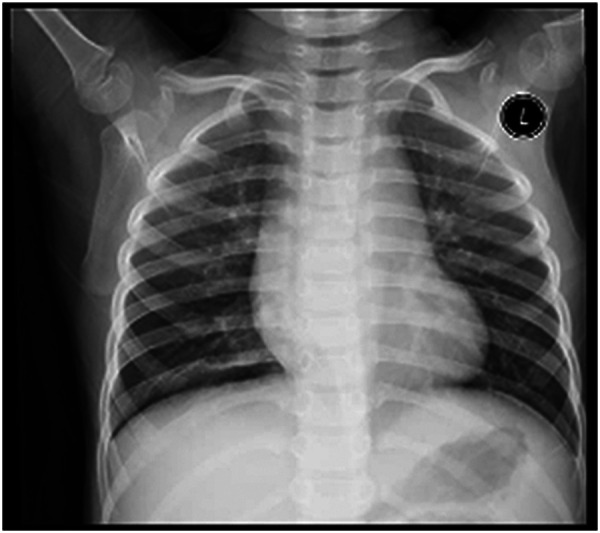
Chest x-ray findings six months into the course of the illness.

A 5-year-old male child presented with fever and cough for 6 days, which persisted for a total of 14 days. The fever occurred 4–6 times daily, with a peak temperature of 40.2°C. The cough was paroxysmal and accompanied by continuous sounds. The patient had a normal appetite and normal bowel and bladder habits. The patient, born from a first pregnancy via full-term cesarean section, weighed 3.95 kg at birth with no history of asphyxia or resuscitation. The patient has been experiencing normal growth and development with no significant family history of hereditary or infectious diseases. At 3 years old, he had an episode of intussusception without a history of recurrent respiratory infections. On admission, the patient's vital signs were as follows: temperature 39.0°C, heart rate 124 beats per minute, respiratory rate 30 breaths per minute, and blood pressure 102/65 mmHg. Physical examination revealed congested throat, increased respiratory effort, mild intercostal retractions, symmetrical chest expansion, increased tactile fremitus in the right lung, dullness on percussion of the right lung, decreased breath sounds on auscultation, and bilateral crackles. The abdomen was mildly distended, and the liver and spleen were palpable, with the liver 1 cm below the costal margin and a sharp edge resembling the spleen. No other abnormalities were noted. Initial diagnosis was severe pneumonia, suspected to be caused by *Mycoplasma pneumoniae* based on the clinical presentation and local epidemiology. Despite outpatient treatment with azithromycin for 6 days, there was no improvement, raising concern for bacterial co-infection. The patient received azithromycin, piperacillin-tazobactam, methylprednisolone sodium succinate, and ambroxol hydrochloride. Upon admission, white blood cell count (WBC) was 8.0 × 10^9^/L with 81.7% neutrophils, elevated C-reactive protein (CRP) 43.7 mg/L. Other findings included elevated erythrocyte sedimentation rate (ESR) 65 mm/h, ferritin 537.7 μg/L (reference range: 22–322 μg/L), lactate dehydrogenase (LDH) 554 U/L, alpha-hydroxybutyrate dehydrogenase (α-HBDH) 414 U/L, prolonged Partial thromboplastin time (55.0 s), fibrinogen elevated (4.98 g/L), and increased D-dimer levels 0.36 mg/L (reference range: 0–0.3 mg/L). Serology showed *Mycoplasma pneumoniae* antibody titers of 1:160, normal immunoglobulins, and T-lymphocyte subset analysis. Liver function tests showed normal. The results from the antibody testing for Hepatitis A, B, and C show a positive outcome for HBsAb, with negative findings for the remaining antibodies. Moreover, both the TP-ELISA screening for syphilis and the HIV antibody test displayed negative results. The assessments for respiratory virus antigens, including Influenza A and B, Adenovirus, Respiratory Syncytial Virus, and Parainfluenza Virus types 1, 2, and 3, all returned negative. Additionally, the Tuberculosis T-Cell Spot Test (T-spot) also presented a negative outcome. Thoracic ultrasound showed a small amount of pleural effusion on the right side (5 mm). Despite treatment, the patient's condition deteriorated after 5 days with persistent fever, unimproved cough, mental deterioration, respiratory distress, and hepatosplenomegaly. Upon reexamination, the blood tests revealed elevated WBC (18.1 × 10^9^/L) with 85.3% neutrophils, increased CRP levels (85.6 mg/L), and findings of vacuolar changes in the blood smear. The ESR was notably high at 99 mm/h, with elevated ferritin levels (688.5 μg/L) and procalcitonin (PCT) levels of 0.618 μg/L. Additionally, LDH and *α*-HBDH levels were elevated at 726 U/L and 529 U/L, respectively. Liver function tests showed elevated alanine aminotransferase (ALT) at 123 U/L and aspartate aminotransferase (AST) at 170 U/L. Serum protein analysis displayed total protein at 60.0 g/L and albumin at 24.1 g/L. Coagulation tests indicated prolonged prothrombin time (34.4 s), prolonged activated partial thromboplastin time (55.0 s), and elevated fibrinogen levels (6.78 g/L). D-dimer levels were elevated at 0.73 mg/L. Abdominal ultrasound revealed hepatosplenomegaly (liver 4 cm below the costal margin) with increased echogenicity of the liver parenchyma, and a small amount of fluid accumulation in the abdominal cavity. Thoracic ultrasound showed increased pleural effusion on both sides (left 25 mm, right 21 mm).The patient exhibited a rise in *Mycoplasma pneumoniae* antibody titers (1:1280). The testing revealed further increases in white blood cell count, decreased albumin levels, and continued pleural and abdominal fluid accumulation necessitating a change in the treatment plan. The elevated titer of *Mycoplasma pneumoniae* antibodies suggests *Mycoplasma pneumoniae* infection. The white blood cell count is higher than before, not excluding the relevance of methylprednisolone sodium succinate. However, the child's condition has worsened with severe inflammatory responses. Although bacterial cultures from sputum and other tests have not detected bacteria, considering the use of antibiotics may impact bacterial detection, bacterial infection is not ruled out. The revised treatment plan includes antibiotics such as teicoplanin and azithromycin, intravenous administration of albumin and immunoglobulin, thoracentesis with drainage of the right pleural cavity, bronchoscopy, and bronchoalveolar lavage. Bronchoscopy and bronchoalveolar lavage showed abundant white, thick, and viscous secretions in the right lower lobe bronchus, which were removed using a brush and washed with diluted N-acetylcysteine and saline (Figure 1). The bronchoalveolar lavage fluid analysis indicated various cell types: neutrophils accounted for 38%, mononuclear lymphocytes for 36%, macrophages for 25%, and eosinophils for 1%. Under high magnification, white blood cells were abundant, with a few showing intracellular granules displaying active movement. The sample was PAS(-) and tested positive for lipid staining, with negative bacterial cultures and pathogens, except for positive results for *Mycoplasma pneumoniae* and drug-resistant *Mycoplasma pneumoniae*. The smear showed no acid-fast bacilli, was negative for ink staining, and revealed no fungi. On the 7th day of the illness, consolidation appeared in the right upper lung, with a subsequent increase in the extent of consolidation in the right middle and lower lung lobes (Figure 2). The symptomatic support treatment including previous anti-infective and anti-inflammatory measures continued. Throughout the infection, the patient's condition gradually improved. By the 15th day of the illness, the patient's temperature normalized. On the 21st day of the illness, the child's temperature began to fluctuate, reaching a peak of 38.2°C. There was no worsening cough, and the child's mental status and appetite remained satisfactory. Repeat blood tests showed: WBC 15.3 × 10^9^/L with 77.9% neutrophils, CRP 3.0 mg/L; ESR was 49 mm/h, ferritin 316.9 μg/L; PCT 0.124 μg/L; liver function tests indicated elevated ALT at 37 U/L and AST at 23 U/L levels. Cardiac enzymes: LDH 317 U/L and *α*-HBDH 264 U/L levels were elevated; serum protein analysis showed total protein at 72.1 g/L and albumin at 33.1 g/L. A follow-up chest x-ray revealed a large dense shadow with uneven density in the right middle and lower lobe of the lung, with multiple cystic lucencies visible at the lower margin (Figure 3). Subsequent lung CT scan showed significant necrosis in the entire right middle and lower lung lobes (Figure 4). Throughout the anti-infective and anti-inflammatory treatment, the child's vital signs were closely monitored. On the 31st day of the illness, the child's temperature normalized, there was no cough, and repeat blood tests showed: WBC 6.4 × 10^9^/L with 52.9% neutrophils, CRP 3.0 mg/L; thoracic ultrasound indicated right pleural effusion (7.7 mm) and partial consolidation in the right lung; abdominal ultrasound showed no significant abnormalities. On the 44th day of illness, the necrotic foci in the right middle and lower lobes decreased, while compensatory enlargement of the right upper lobe occurred (Figure 5). On the 72nd day of illness, further reduction of necrotic lesions in the right middle and lower lobes was observed (Figure 6). Follow-up assessments on the 44th and 72nd days demonstrated a reduction in lung necrosis and improvement in necrotizing pneumonia caused by drug-resistant *Mycoplasma pneumoniae* infection, with complications including pleural effusion, ascites, coagulation and liver dysfunction, and hypoalbuminemia. Treatment lasted approximately 2.5 months, with a follow-up at six months showing minimal residual cystic densities in the right lower lung (Figure 7).

## 3 Discussions

In this case, a 5-year-old male child presented with a 14-day history of fever and cough. The patient exhibited several clinical manifestations, including paroxysmal cough with continuous sounds, congested throat, increased respiratory effort, intercostal retractions, and abnormal lung findings on physical examination. Laboratory investigations revealed various abnormalities, such as elevated white blood cell count, increased inflammatory markers (e.g., C-reactive protein), elevated liver enzymes, coagulation dysfunction, and hypoalbuminemia. Imaging studies, including thoracic ultrasound and pulmonary CT scans, showed the presence of pleural effusion and progressive necrotizing pneumonia.

*Mycoplasma pneumoniae* is a significant global cause of community-acquired pneumonia (1). It accounts for a considerable proportion of pneumonia cases, including those that result in severe complications such as necrotizing pneumonia (2). The epidemiology of *Mycoplasma pneumoniae* infection varies across different regions and populations. In China, *Mycoplasma pneumoniae* is a common etiological agent of pneumonia, particularly among pediatric patients (3). A search on PubMed using the keywords “necrotizing pneumonia and Mycoplasma pneumonia” yielded 57 articles, while a search using the keywords “necrotizing pneumonia and Mycoplasma pneumonia and China” yielded 20 articles. Based on the available data, approximately 35% (20/57) of the articles retrieved from the search were related to China. This indirectly indicates a higher incidence of *Mycoplasma pneumoniae* pneumonia in China and potentially a higher occurrence of cases progressing to necrotizing pneumonia.

The possible Factors of necrotizing pneumonia caused by *Mycoplasma pneumoniae* infection can be discussed. Several studies, including those by Yonghan Luo (4) and Baoying Zheng (5), have reported similar findings of prolonged fever, increased lactate dehydrogenase (LDH) levels, and elevated C-reactive protein (CRP) levels in patients with necrotizing pneumonia caused by *Mycoplasma pneumoniae* infection. In this case, the patient experienced a fever lasting for 14 days, along with elevated LDH and CRP levels, which aligns with the findings of Yonghan Luo and Baoying Zheng. Additionally, the patient's D-dimer showed a more than two-fold increase, although not as significant (5). This could be an isolated case or possibly influenced by the timing of the D-dimer test. It highlights the importance of monitoring D-dimer levels throughout the course of the illness in pediatric patients. Furthermore, the absence of significantly elevated D-dimer levels does not exclude the possibility of necrotizing pneumonia.

Xia Huang's (6) study classified the imaging manifestations of *Mycoplasma pneumoniae* pneumonia into different categories, such as bronchopneumonia, consolidation/atelectasis, bronchitis, and nodular changes. Patients in the consolidation/atelectasis group have a poorer prognosis and a higher likelihood of developing necrotizing pneumonia. In this case, the initial imaging showed consolidation in the right middle and lower lobes, which is consistent with Xia Huang's findings. Xia Wang's (7) study revealed that 80.0% of patients with *Mycoplasma pneumoniae*-induced necrotizing pneumonia developed pleural effusion, which was also observed in this case. The study (7) further indicated that the average duration from symptom onset to the discovery of necrotic lesions in *Mycoplasma pneumoniae* pneumonia is 21.0 ± 6.9 days, and chest lesions usually resolve or show minimal residual fibrotic changes within 3.0 (2.0–6.0) months. In this case, the necrotic lung lesions were observed on day 21, decreased on day 44 with compensatory enlargement of the normal right upper lobe, and significantly reduced on day 72. However, it is important to note that the necrotic lung tissue in the right middle and lower lobes did not fully recover but compensated through the normal lung tissue of the upper lobe. This is an uncommon occurrence, as severe necrotic lung lesions caused by *Mycoplasma pneumoniae* infection are rarely observed.

In conclusion, this case highlights the clinical presentation, laboratory findings, and radiographic manifestations of a 5-year-old male child with necrotizing pneumonia caused by *Mycoplasma pneumoniae* infection. The prolonged fever, elevated LDH and CRP levels, as well as the presence of pleural effusion and necrotic lung tissue, are consistent with previous studies. Monitoring D-dimer levels is essential, even in the absence of a significant increase, to evaluate the possibility of necrotizing pneumonia. The resolution time of chest lesions may vary, and in rare cases, compensatory enlargement of the normal lung tissue can occur.

Here is a simplified version of the parental perspective: Early in the child's illness, after 5 days of treatment, the family became concerned as their child didn't improve like other children in the ward. Worries about the treatment plan and potential risks arose. Subsequent improvements and a lung CT scan revealing necrotizing pneumonia heightened anxieties. They considered if earlier treatment at a better hospital could have helped. Currently, the child is well with no respiratory issues. Trust in the medical team and active participation were crucial. Sharing this experience may help others in similar situations.

Upon review, it's clear that the child's diagnosis and treatment were thorough. Despite standard protocols, necrotizing pneumonia developed, likely due to *Mycoplasma pneumoniae* resistance and the child's health. Future consideration could involve early high-dose corticosteroids to prevent such complications. The family's experience highlights the emotional challenges caregivers face, emphasizing the crucial role of communication, trust, and collaboration between medical teams and families in achieving the best outcomes.

## Data Availability

The original contributions presented in the study are included in the article/Supplementary Material, further inquiries can be directed to the corresponding author.
